# Self-Assembly Processes in Hydrated Montmorillonite by FTIR Investigations

**DOI:** 10.3390/ma13051100

**Published:** 2020-03-02

**Authors:** Maria Teresa Caccamo, Giuseppe Mavilia, Letterio Mavilia, Domenico Lombardo, Salvatore Magazù

**Affiliations:** 1 Dipartimento di Scienze Matematiche e Informatiche, Scienze Fisiche e Scienze della Terra, Università di Messina, Viale Ferdinando Stagno D’Alcontres n 31, S. Agata, 98166 Messina, Italy; mcaccamo@unime.it (M.T.C.); gmavilia1@unime.it (G.M.); 2 Dipartimento di Patrimonio, Architettura e Urbanistica; Università Mediterranea di Reggio Calabria, (PAU), Via Melissari, I-89124 Reggio Calabria, Italy; letterio.mavilia@unirc.it; 3 CNR-IPCF, Viale Ferdinando Stagno d’Alcontres 37, 98158 Messina, Italy; lombardo@ipcf.cnr.it

**Keywords:** self-assembly processes, montmorillonite, IR spectroscopy, OH stretching band, wavelet Analysis

## Abstract

Experimental findings obtained by FTIR and Raman spectroscopies on montmorillonite-water mixtures at three concentration values are presented. To get some insight into the hydrogen bond network of water within the montmorillonite network, FTIR and Raman spectra have been collected as a function of time and then analyzed following two complementary approaches: An analysis of the intramolecular OH stretching mode in the spectral range of 2700–3900 cm^−1^ in terms of two Gaussian components, and an analysis of the same OH stretching mode by wavelet cross-correlation. The FTIR and Raman investigations have been carried as a function of time for a montmorillonite-water weight composition (wt%) of 20–80%, 25–75%, and 35–65%, until the dehydrated state where the samples appear as a homogeneous rigid layer of clay. In particular, for both the FTIR and Raman spectra, the decomposition of the OH stretching band into a “closed” and an “open” contribution and the spectral wavelet analysis allow us to extract quantitative information on the time behavior of the system water content. It emerges that, the total water contribution inside the montmorillonite structure decreases as a function of time. However, the relative weight of the ordered water contribution diminishes more rapidly while the relative weight of the disordered water contribution increases, indicating that a residual water content, characterized by a highly structural disorder, rests entrapped in the montmorillonite layer structure for a longer time. From the present study, it can be inferred that the montmorillonite dehydration process promotes the layer self-assembly.

## 1. Introduction

Among various silico-aluminate precursors used for the preparation of geopolymers, we can certainly mention clays and in particular those characterized by cation exchange, such as montmorillonite. The most used cation exchange clays are those belonging to the class of layered phyllosilicates. Phyllosilicates are clay minerals, readily available, characterized by a lamellar structure consisting of two-dimensional layers (i.e., lamellae) with spaces between the various lamellae containing cations and water molecules. Each lamella is formed by the union of individual layers of silica with tetrahedral coordination, joined to layers of alumina or magnesia with octahedral coordination [1,2,3,4].

**Figure 1:**
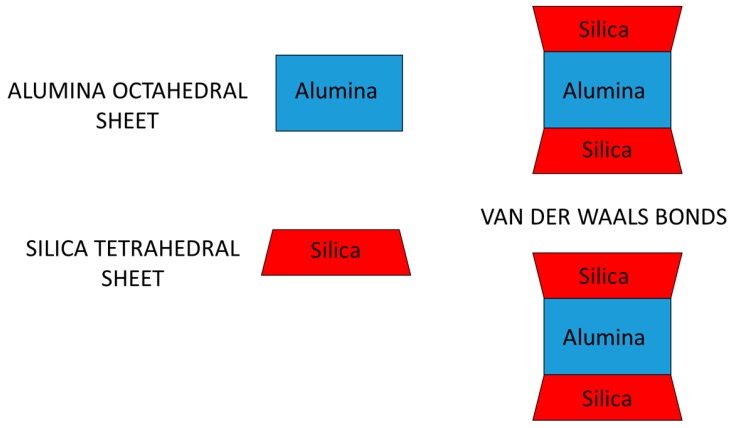
Layered structure of montmorillonite with a 2:1 ratio, in which a single lamella is formed by two silica tetrahedral sheets with an interposed alumina octahedral sheet.

There are two types of phyllosilicates: Those having a 1:1 ratio, such as kaolin, in which each lamella is made up by only two layers (one octahedral and one tetrahedral) and the phyllosilicates with a 2:1 ratio, such as montmorillonite, in which a single lamella is formed by an octahedral layer interposed between two tetrahedral layers and, moreover, there is the presence of weak Van der Waals bonds between the various lamellae. Figure 1 shows, in a schematic way, the layered structure of montmorillonite.

The montmorillonite chemical formula is (Si^7.8^Al^0.2^)^IV^(Al^3.4^Mg^0.6^)^VI^O_20_(OH)_4_; its composition, without considering the presence of the material between the various lamellae, is: SiO_2_ (66.7%), Al_2_O_3_ (28.3%), H_2_O (5%), which allows us to explain how in the montmorillonite there may be isomorphic substitutions of the Si^4+^ cations with Al^3+^ within the tetrahedral units, and of the Al^3+^ cation with Mg^2+^ in the octahedral units. In this way, the montmorillonite net charge for each layer of is: [7.8 (+4)] + [0.2 (+3)] + [3.4 (+3)] + [0.6 (+2)] + [20 (−2)] + [4 (−1)] = −0.8 charge/unit cell [5,6,7]. Thus, the lamellae constituted by these layers are characterized by an excess negative charge, which is balanced by the elements in the interlamellar spaces such as alkaline or alkaline-earth cations, solvated, in turn, by water molecules. Montmorillonite is a material highly available in nature: It constitutes, in fact, the main component of bentonite, representing 50% of it.

**Figure 2:**
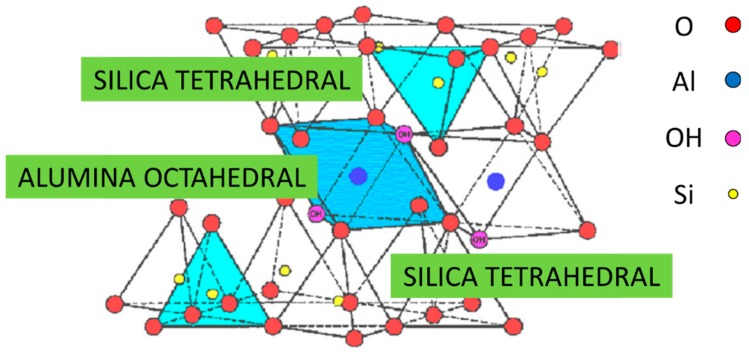
Crystallographic structure of montmorillonite, which is composed by a central alumina octahedral sheet within two silica tetrahedral sheets, forming a common layer [5].

In addition, montmorillonite crystallizes in lamellae with a thickness of nanometric sizes, characterized by a surface length that can reach the micrometric sizes, forming aggregates composed of about 25 overlapping lamellae. This feature gives montmorillonite a very high surface/volume ratio (250–800 m^2^/g versus 10 m^2^/g of talc). The crystallographic structure of montmorillonite (Figure 2) is, as also partially anticipated and represented previously in Figure 1, a 2:1 layered structure of silica and alumina: A single octahedral layer of alumina (Al_2_O_3_) is therefore observed between two tetrahedral layers of silica (SiO_4_), and so the oxygen ions of the octahedral layer also belong to the tetrahedral layer [8,9].

The number of terminal oxygenals of the silicate layers is often not sufficient to complete the octahedral coordination, and therefore the remaining vertices are occupied by additional OH^−^ ions. Lamellar silicate is characterized by having only two thirds of the available octahedral sites occupied: The cations that occupy them are Al^3+^. They are isomorphically replaced by Mg^2+^ and Fe^2+^ cations and, in addition, there are also Al^3+^ cations in place of the tetrahedral silicon cations. The excess of negative charge that is created is balanced by various mono or bivalent cations (Ca^2+^, Mg^2+^, Na^+^, K^+^) solvated and which, being unable to be inserted inside the crystal, remain located on the edges of the same, occupying the interlayer region [10,11,12].

Cations and water are not part of the crystalline structure and can be easily replaced by other cations: The lamellae layers are bonded each other by weak interaction forces (Van der Waals bonds) and therefore can be easily separated from each other [13,14].

As far as functional properties are concerned, montmorillonites are characterized by significant Cation Exchange Properties (C.E.C.): The values of C.E.C. for montmorillonite vary between 80 and 120 meq/100 g depending on the degree of isomorphic substitutions occurred in the lattice (Table 1).

Another important property of montmorillonite, in addition to the cation exchange capacity evaluated by C.E.C., is that related to the expansion capacity of this molecular structure linked to the presence of H_2_O molecules between one layer and another. This expansion also occurs in the presence of polar liquids such as ethylene glycol and glycerin [15,16].

**Figure 3:**
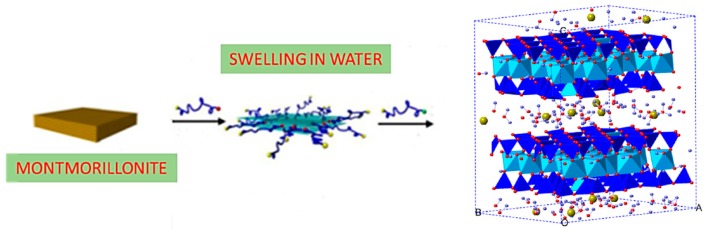
Swelling of montmorillonite in water [19].

Furthermore, the distance between the reticular planes of montmorillonite depends on the degree of hydration of the mineral: By increasing the number of water layers, the crystalline lattice expands (Figure 3); by complete dehydration, however, it loses its ability to expand. In fact, in the presence of water, H_2_O molecules dispose themselves within the montmorillonite interlayer space, generating a space increase between the layers. In aqueous solutions, montmorillonite aggregates can disassociate, giving rise to montmorillonite nanolayers [17,18].

Such disassociation depends on the montmorillonite concentration, and on the nature of the cations in the interlayer space [19]. As a rule, the higher the cationic charge is, the larger the particle size are.

In the case of Na^+^-Montmorillonite, when water is added to powder, Na^+^ ions hydrate; in such a case, a huge number of H_2_O molecules penetrate within the interlayer space; due to such a penetration, the montmorillonite crystalline structure expands along a direction which is perpendicular to the layers.

In the case of Ca^2+^-Montmorillonite, instead, when water is added to powder, the Ca^2+^ ions remain within the space between the layers and on the hydroxyl sites located within the layers. In order to increase the montmorillonite exfoliation process in solution, ultrasonication is often used [20,21,22,23].

Another peculiar characteristic of montmorillonite is thixotropy, that is, that peculiar characteristic of a few clays to give rise, when they are dispersed in a certain aqueous solution with appropriate concentration to colloidal suspensions, which remain in the liquid state (sol) if stirred; however, when at rest, they take on a certain consistency and viscosity (gel).

Other important physical properties of montmorillonite are reported in Table 2 [4]. 

In addition, among other functional properties of montmorillonite, one can mention electrical conductivity (mS/m), whose value, being this clay characterized by an intrinsic porosity, depends on the conductivity of the fluid that passes through these pores [24,25,26]. Furthermore, as far as thermal properties are concerned, montmorillonite is a good thermal insulator. As far as the water absorption capacity, this feature is very important for these natural clays. In fact, clays can absorb or desorb water as a function of changes in the moisture content: As above reported, when H_2_O molecules are absorbed, they fill the space between the various layers [27]. Montmorillonite has excellent water absorption properties; however, the interaction between the water molecules and montmorillonite can cause swelling. The absorption of water molecules and the swelling of montmorillonite leads to the formation of hydrated states and can give rose to hysteresis phenomena. Ionic migration towards the central plane between the layers, determines the phenomenon of montmorillonite swelling [28]. The montmorillonite swelling and hydration processes play a key role for a wide variety of engineering applications. Moreover, the anisotropy of a wide class of clay minerals is reflected in a wide variable range of mechanical properties. The structure of hydrated Na-montmorillonite is shown in Figure 3. Molecular Dynamic (MD) simulations performed by Zheng and A. Zaoui [29] showed that the elastic constants of dried and hydrated Na-MNT are different. The anisotropy of Na-montmorillonite can generate great differences in the values of elastic constants, bulk, and shear modulus, and Young’s modulus. These mechanical quantities decrease with increasing hydration. 

Montmorillonite is used as both silico-aluminate precursor to be subjected to alkaline activation for the production of geopolymers and as nanofiller for the production of nanocomposite materials used in the food and biomedical fields with high antibacterial properties. In addition, they can be used as adsorbent materials and as catalysts in green chemistry. Recent researches employ the self-assembling methods between supramolecular objects to fabricate innovative well-defined nanomaterials that link soft matter approaches to hard matter components. For example, the soft-templated mesoporous nanomaterials allow to construct nanomaterials with controllable structure and properties, and require the employment of techniques to simultaneously detect the structure–structure re-organization and dynamics at the nanoscale [30,31,32,33,34]. In the present work, we have not considered the acid activation process of montmorillonite. In this experimental work, FTIR and Raman investigations on montmorillonite water mixtures as a function of time are carried out in order to follow the montmorillonite dehydration process, as well as to characterize the process of montmorillonite layer self-assembly.

## 2. Materials and Methods

Pure powder montmorillonite purchased from Merck (Milano, Italy, surface area 250 m^2^/g) and double distilled water were employed to prepare the samples. Three samples of hydrated montmorillonite were prepared by weight at three concentration values: (i) 20 wt% of montmorillonite and 80 wt% of water; (ii) 25 wt% of montmorillonite and 75 wt% of water; and 35 wt% of montmorillonite and 65 wt% of water. The samples were then mixed and then treated in order to get a 1mm thick layer of hydrated montmorillonite.

Fourier Transform Infrared (FTIR, Bruker Optics, Ettlingen, Germany) spectroscopy allows us to characterize the molecule rotational and vibrational motions [35,36,37,38,39,40]. Such a spectroscopic technique explores 14000–10 cm^-1^ range of the e-m spectrum, which encompasses the Near-IR (NIR) range (14000–4000 cm^−1^), the Mid-IR (MIR) range (4000–400 cm^−1^), and the Far- IR (400–10 cm^−1^). FTIR is, in some regards, a complementary technique in respect to other techniques such as Raman and inelastic neutron spectroscopies and density function simulations [28,41,42,43,44,45,46,47,48]. In the present study, the FTIR study was carried out in the range 400–4000 cm^−1^ with a spectral resolution of 4 cm^−1^.

Furthermore, Raman spectra were registered by the spectrometer BRAVO (Bruker Optics), operating in the 450–3200 cm^−1^ range. The source was constituted by two lasers working at the wavelength of 785 nm and 1064 nm. The explored spectral range was 300–3200 cm^−1^. The spot size was 10–15 micron at 10× lens.

## 3. Analysis and Discussion

In Table 3, the FTIR spectral features of the hydroxyl groups associated with octahedral cations, quartz, silicates, and water are listed. The most intense bands are at 1035 cm^−1^ (stretching in the Si-O plane) and at 529 cm^−1^ (Si−O bending vibration). The vibration at 1113 cm^−1^ represents the stretching out of the Si–O plane. The wide bands at 3440 and 1639 cm^−1^ represent the stretching and bending vibrations of the OH water molecules.

**Figure 4:**
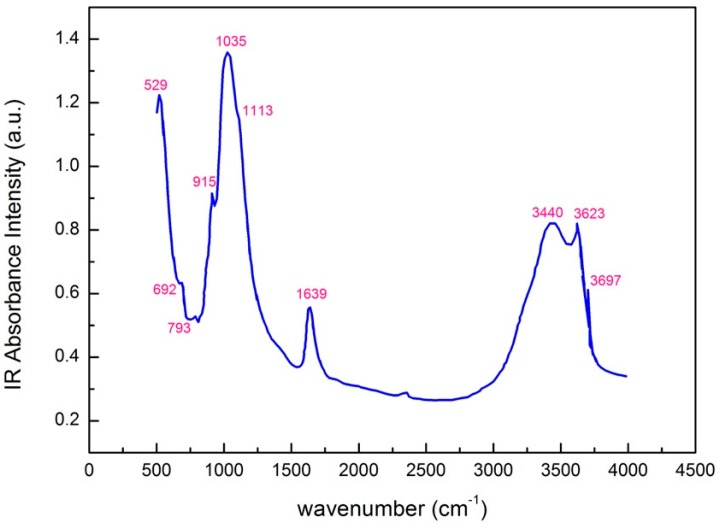
FTIR spectrum of pure montmorillonite showing the characteristic bands and vibration frequencies.

The FTIR spectrum of pure montmorillonite (Figure 4) shows bands located at 3697 and 3623 cm^−1^, which are attributed to the OH groups coordinated with the octahedral cations, including the vibration at 3623 cm^−1^, which is due to the OH group bound with Al^3+^ cations.

**Figure 5:**
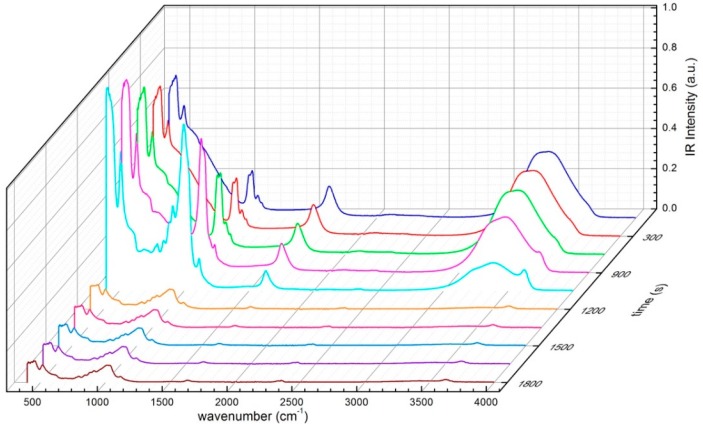
FTIR spectra of montmorillonite-water mixtures, for the concentration of 25 wt% of montmorillonite; and 75 wt% of water, in the 400 < ∆ω < 4000 cm^−1^ spectral range vs. time.

The 3623 cm^−1^ band indicates the substitution of octahedral Al^3+^ by Fe^2+^ or Mg^2+^ cations. As far as the hydrated samples are concerned, in the present work we address the attention to the OH stretching band, which interests the frequency range of 2700–3900 cm^−1^. In particular, the FTIR spectra are observed as a function of time (Figure 5), and then deconvoluted into Gaussian components; finally, their time dependence is discussed [49,50,51,52,53,54,55,56,57]. For a comparison, the spectral profiles were normalized. It should be noticed that a typical FTIR spectrum is characterized by two major bands; by lowering temperature, the low-frequency band, centered at 3200 cm^−1^, becomes more prominent in respect to the high frequency peak at 3400 cm^−1^, while the total spectrum shifts towards lower frequencies; at the same time, in presence of a dehydration process, it is expected that at first the water open contribution, corresponding to bulk water decreases, whereas the so called water closed contribution, connected with bound water persists for a longer time [58,59,60,61,62]. However, it should be noticed that the spectrum could be deconvoluted into five Gaussians centered at 3050, 3200, 3400, 3500, and 3650 cm^−1^. However, in the present study, we will adopt a two-state model [63,64,65,66]. To describe the structure and thermodynamic properties of liquid water within the montmorillonite matrix, we adopt the so-called two-state model [67,68], which assumes two different states of intermolecular bonding: One is an ice-like state where water molecules are more ordered, and another one is a more densely packed arrangement where hydrogen bonds are distorted [69,70,71]. Here, we apply the two-state model and we partition the H_2_O molecules into: (i) H_2_O molecules with two OH groups both hydrogen-bonded to a tetrahedral network, and (ii) H_2_O molecules whose hydrogen bond is broken or weakened by distortion.

**Figure 6:**
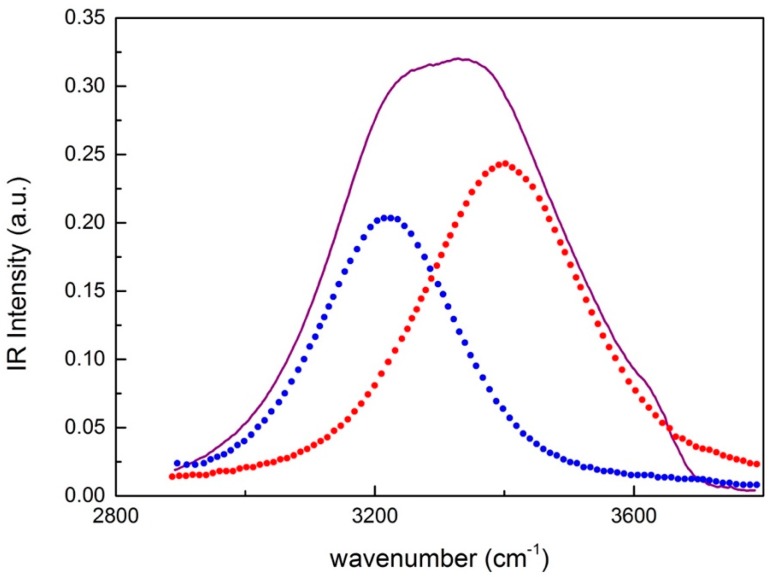
Decomposition of the intramolecular contribution into a Gaussian band corresponding to “open” water (blue dots) and a Gaussian band corresponding to “closed” water (red points).

Before the analysis, spectra of pure montmorillonite were subtracted from the spectra of hydrated montmorillonite. Therefore, considering the spectra of the O–H stretching band for montmorillonite-water mixtures at different times, it is possible to decompose the spectra into a ‘closed’ and an ‘open’ contribution; the open contribution ($\sim 3200\;{\mathrm{cm}}^{-1}$) is attributed to the O–H vibrations in tetrabonded H_2_O molecules, while the closed contribution ($\sim 3400\;{\mathrm{cm}}^{-1}$) can be attributed to the O–H vibration of H_2_O molecules with a not fully developed hydrogen bond. Figure 6 shows the decomposition of the intramolecular contribution into a Gaussian band corresponding to “open” water and a Gaussian band corresponding to “closed” water.

**Figure 7:**
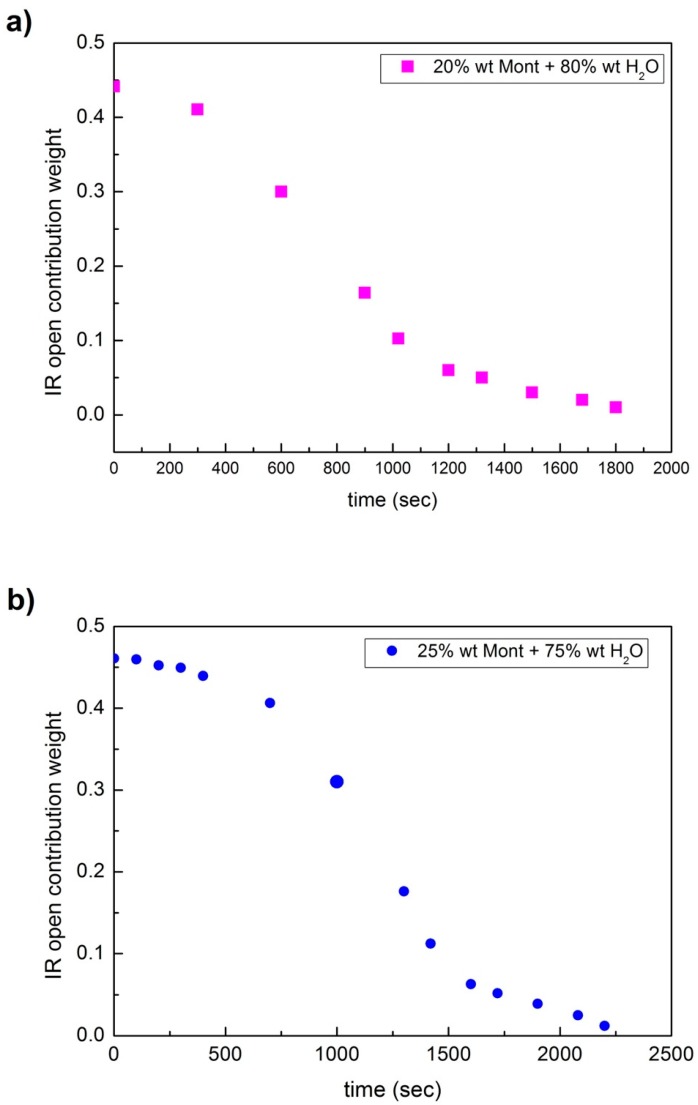

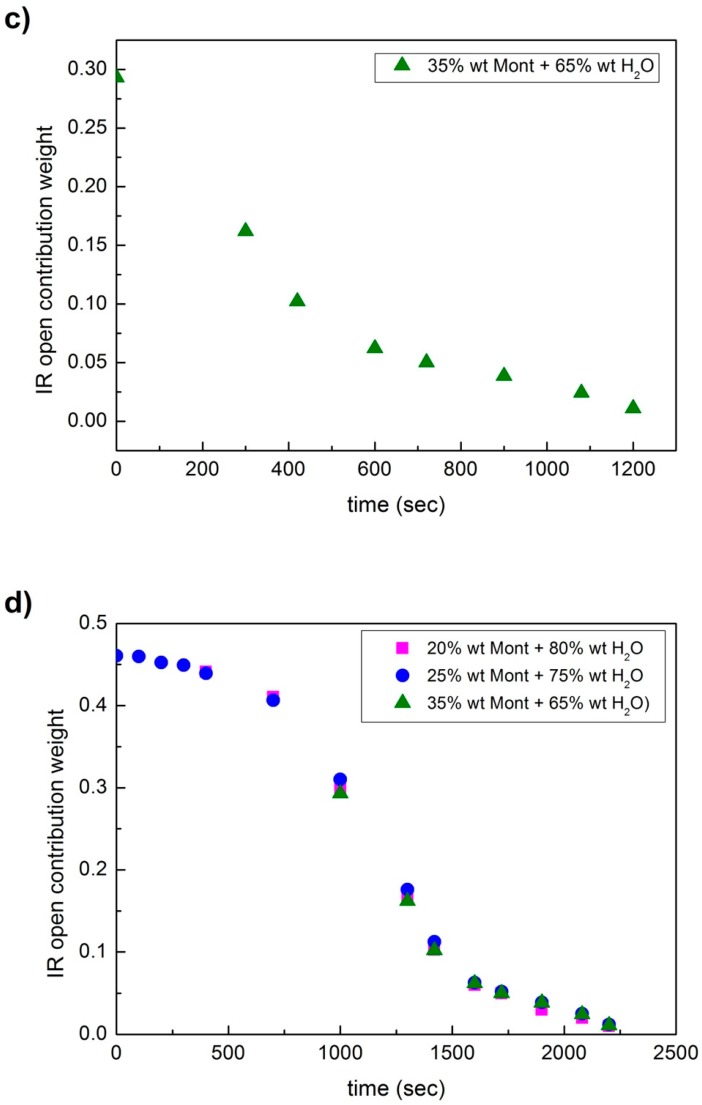
Behavior of the weights of the open contribution as a function of time for a concentration value of: (**a**) 20 wt% of montmorillonite and 80 wt% of water; (**b**) 25 wt% of montmorillonite; and 75 wt% of water; and (**c**) 35 wt% of montmorillonite and 65 wt% of water; finally, (**d**) data obtained starting from the three different concentration values, scaled along the time axis; as it can be seen, the data follow the same trend.

Figure 7 reports the behavior of the weights of the open contribution as a function of time for a concentration value of: (a) 20 wt% of montmorillonite and 80 wt% of water; (b) 25 wt% of montmorillonite; and 75 wt% of water; and (c) 35 wt% of montmorillonite and 65 wt% of water. Figure 7d shows the data obtained starting from the three different concentration values, scaled along the time axis. As it can be seen, the data arrange along the same trend. What emerges is that while the total water contribution decreases in time with a characteristic time of 1093 s, determined by the inflection point of the fitting curve, the relative weight of the open contribution decreases faster with a characteristic time of 734 s; in the meantime, the relative weight of the closed contribution, in respect to the open contribution, increases.

To characterize the montmorillonite-water interaction, an innovative wavelet cross correlation technique has been employed [72,73,74,75,76,77,78]. In particular, such a method allows us to identify the degree of similarity between two individual spectra. Furthermore, this method has been employed in different scientific domains, like physics, mathematics, climate, geoscience, and engineering science [79,80,81,82,83,84,85,86,87,88]. The wavelet cross-correlation coefficient, $r_{XWT}$, takes into account the two wavelet transforms, $w_{1}\left(a,\tau \right)$ and $w_{2}\left(a,\tau \right)$, of the considered spectra and the two wavelet spectra $S_{1}\left(a\right)$ and $S_{2}\left(a\right)$ [89,90,91,92,93,94,95,96,97,98,99]. More precisely, a wavelet transform wt(a,τ) is the inner product of the function f(x) with translated and scaled mother wavelets ψ:

(1) $$w\left(a,\tau \right)=\frac{1}{a}\int_{-\infty }^{+\infty }f\left(x\right)\psi^{*}\left|x-\tau \right|dx;$$

where a denotes the scale parameter (a>0), τ represents the shift parameter, f(x) is the one-dimensional function, * characterizes the complex conjugate, and ψ is the mother wavelet:

(2) $$\psi_{a,\tau }=\frac{\psi \left(x-\tau \right)}{a};$$

by these expressions, one defines the wavelet spectrum S(a):

(3) $$S\left(a\right)=\frac{1}{a}{\left|w\left(a,\tau \right)\right|}^{2}dx;$$

Finally, one determines the wavelet cross-correlation coefficient, $r_{XWT}$:

(4) $$r_{XWT}\left(a\right)=\frac{\int w_{1}\left(a,\tau \right)w_{2}^{*}\left(a,\tau \right)d\tau }{\sqrt{S_{1}\left(a\right)S_{2}\left(a\right)}}$$

**Figure 8:**
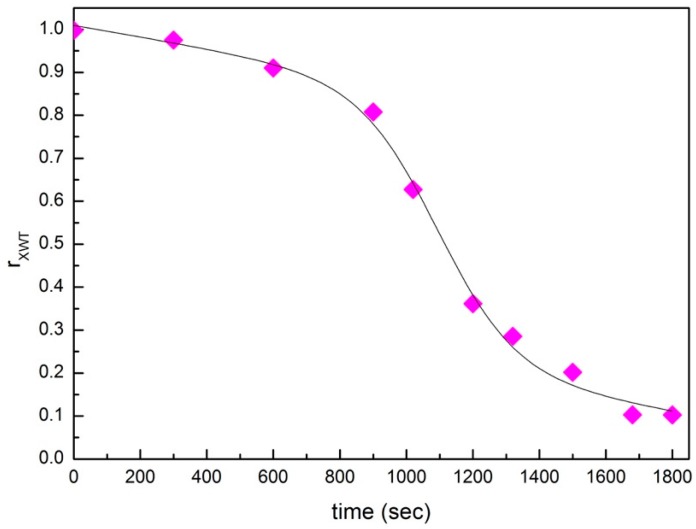
Wavelet cross-correlation coefficient, $r_{XWT}$ as a function of time for the concentration of 25 wt% of montmorillonite; and 75 wt% of water.

In this work we choose as reference wavelet spectrum the first spectrum at time 0 s. Figure 8 reports the evaluated wavelet cross-correlation coefficient, $r_{XWT}$, as a function of time for the concentration of 25 wt% of montmorillonite; and 75 wt% of water [100,101,102,103,104,105]. It clearly emerges from the figure that the wavelet cross-correlation coefficient $r_{XWT}$ decreases as a function of time following a decreasing sigmoid behavior whose inflection point abscissa is in excellent agreement with the value extracted from the behavior of the open contribution weight as a function of time (see Figure 7).

As far as the Raman measurements are concerned, the spectral features of pure montmorillonite clay are reported in Table 4. The assignments agree with those reported in [106]. In montmorillonite, the central octahedral atom substitution can be inferred from the spectral region of 785–915 cm^−1^; the AlMgOH spectral band is located at 840 cm^−1^, the AlFeOH spectral band is located at 890 cm^−1^, and the AlAlOH spectral band is located at 915 cm^−1^.

**Figure 9:**
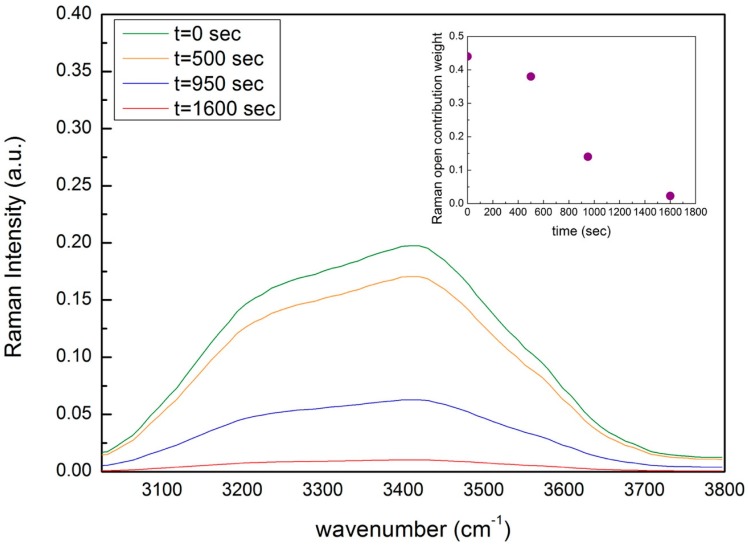
Raman OH stretching contribution as a function time for the concentration value of 25 wt% of montmorillonite and 75 wt% of water. In the insert, the behavior of the weights of the water open contribution vs. time is reported.

Before the analysis, spectra of pure montmorillonite were subtracted from the spectra of hydrated. Figure 9 reports, for the concentration value of 25 wt% of montmorillonite and 75 wt% of water, the Raman OH stretching contribution as a function time, while in the insert, the behavior of the weights of the water open contribution as a function of time is reported. In agreement with FTIR data, while the total water contribution decreases as a function of time, the relative weight of the open contribution decreases faster; in the meantime, the relative weight of the closed contribution, in respect to the open contribution, increases. Furthermore, the inflection point for Raman data coincides with that obtained for the same concentration value with FTIR data.

These behaviors are due to the global decrease of the water content in the montmorillonite-water mixtures, and to the fact that while the tetrahedral contribution connected with free water decreases faster in respect to the closed contribution, being this latter connected to water bonded to the montmorillonite surface.

## 4. Conclusions

FTIR and Raman data have been collected for montmorillonite-water mixtures, at a starting weight composition (wt%) of 20–80%, 25–75%, and 35–65%, until the dehydrated state where the samples appear as a homogeneous rigid layer of clay. To get some insight into the water hydrogen bond network in the presence of montmorillonite, FTIR and Raman spectra have been analyzed following two alternative approaches. In particular, the intramolecular OH stretching mode in the frequency range of 2700–3900 cm^−1^ has been deconvoluted into two Gaussian bands, while the same spectral contributions were analyzed by means of a wavelet cross-correlation approach. The two procedures suggest that the total water contribution decreases in time, with a characteristic time of 1093 s at room temperature. The percentage of the open water contribution decreases in time faster, whereas the percentage of the closed water contribution, in respect to the open contribution, increases, so indicating that a residual water content, characterized by a higher structural disorder, rests entrapped in the montmorillonite layer structures for a longer time, promoting the self-assembly of montmorillonite layers.

## Figures and Tables

**Table 1:** Specific surface and Cation Exchange Properties (C.E.C.) values of montmorillonite.

Mineral	Specific Surface (m^2^ g^−1^)	C.E.C. (meq/100 g)
kaolinite	10 ÷ 20	3 ÷ 10
illite	80 ÷ 100	20 ÷ 30
montmorillonite	250 ÷ 800	80 ÷ 120
chlorite	80	20 ÷ 30

**Table 2:** Physical properties of montmorillonite.

Property Name	Specific Surface (m^2^/g)
Density	2–3 g/cm^3^ (measured)
Molecular weight	36,031 g/mol
Crystal system	Monoclinic
Hardness	1–2 on Mohs scale
Transparency	Translucent
Color	White, green, yellow, pink, red

**Table 3:** FTIR band assignments for montmorillonite clay.

Wavenumber (cm^−1^)	Assignments
3697	O–H stretching
3623	O–H stretching
3440	O–H stretching, hydration
1639	O–H bending, hydration
1113	Si–O stretching, out-of-plane
1035	Si−O stretching, in-plane
915	AlAlOH bending
793	Tridymite (platy forms)
692	SiO_2_ (Quartz)
529	Si–O bending

**Table 4:** Raman band assignments for montmorillonite clay.

3620 cm^−1^	υ(OH) structural OH groups
1110 cm^−1^	υ(SiO) asymmetric mode of SiO_4_ tetrahedron
915 cm^−1^	δ(OH) bonded with octahedral cations; AlOH; wagging mode
840 cm^−1^	δ(OH) bonded with octahedral cations; MgAlOH; wagging mode
785 cm^−1^	δ(OH) bonded with octahedral cations; AlOH; wagging mode
710 cm^−1^	δ(SiO) symmetric mode (A1) of SiO_4_ tetrahedron
430 cm^−1^	δ(OH) libration of OH
